# Bio-grout based on microbially induced sand solidification by means of asparaginase activity

**DOI:** 10.1038/srep16128

**Published:** 2015-11-03

**Authors:** Mengmeng Li, Qing-Long Fu, Qiuzhuo Zhang, Varenyam Achal, Satoru Kawasaki

**Affiliations:** 1 School of Ecological and Environmental Sciences, East China Normal University Shanghai 200241, China; 2 Key Laboratory of Soil Environment and Pollution Remediation, Institute of Soil Science, Chinese Academy of Sciences, Nanjing, China; 3 Faculty of Engineering, Hokkaido University, Sapporo 0608628, Japan

## Abstract

Bio-grout, a new ground improvement method, has been recently developed to improve the mechanical properties, decrease the permeability of porous materials, reinforce or repair cementitious materials and modify the properties of soil or sand. Bio-grout production depends on microbially induced calcite precipitation (MICP), which is driven mainly by an enzyme, urease. However, urease-based MICP process produces excessive ammonia, in addition to secondary pollution generated by urea that is used as substrate in it. In the present study, we reported asparaginase-based MICP process for sand bio-grout development using *Bacillus megaterium*, and results were also compared with urease-based bio-grouts. The asparaginase activity led to significantly less ammonia production compared to urease without compromising with desired properties of a novel grout. The UCS of bio-grout was obtained at 980 kPa, while the permeability was decreased substantially. The mineralogical composition of precipitated substance was identified as calcite using XRD and the crystal morphology was observed under SEM. The mass percentage of calcite in bio-grout was calculated by thermogravimetric analysis and XCT verified calcite precipitation in it. The results confirmed that biocalcification by means of bacterial asparaginase is a potential solution for geotechnical problems. The asparaginase-based MICP process could be of wider acceptance in future.

The importance of old buildings restoration is growing in the construction sector as problems such as detachments, cracking and crystallization of soluble salts, among others, appear or are accentuated^1^. Such problems can be solved through improving mechanical properties of system that can fill cavities, fissures, voids or cracks by development of grouts. The application of grout materials and grouting technique are widely popular for construction nowadays^2^; however, environmental safety must be taken in account while selecting grouts due to negative effects of chemical grouts on environment. In addition, though many grouts were reported with high strength, the compressive strength of grout-injected walls was not found to directly proportional to the compressive strength of the injection grouts used for grouting^3^. Moreover, the grout strength depends mainly on the bond between grout and *in situ* material, which is not necessarily proportional to the compressive or tensile strength of the grout^4^. To achieve this dual nature of novel grout, we should look for such alternative, which is environmentally sound as well as having self-binding property. Researchers are looking for ideal grout that can be used with high injectability and bonding properties. Bio-grout has potential to increase the cohesion with adjacent materials (whether cementitious, sand, stone or soil) that could enhance the mechanical behavior of the whole system. Such grouts can serve as excellent alternative for natural stone also.

Recently, bio-grout has been developed based on biological activity as a new ground improvement technology with potential to use with cementitious materials^5^. It could improve the mechanical properties (strength, stiffness, cohesion, friction), decrease the permeability of porous materials, reinforce or repair cementitious materials and modify the properties of soil or sand^6^,^7^,^8^,^9^. Bio-grout mainly depends on microbially induced calcite precipitation (MICP), carried out by urease producing bacteria. During this process, bacteria hydrolyze a substrate, urea by producing an enzyme, urease that leads to increment in pH by the production of ammonium ions. Further, CO_2_ dissolution takes place and carbonate ions combines with calcium ions either present in the proximal environment or on the cell wall of bacterial cells to precipitate CaCO_3_. The overall reactions may summarize as follow^10^,^11^:


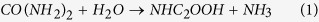



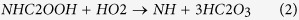



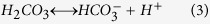



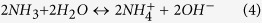



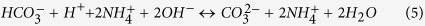



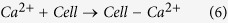



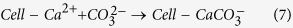


The reactions 6 and 7 may be replaced with following equation.


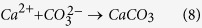


The problem associated with urease driven MICP process is the production of excessive ammonia, which has a negative impact on human health and the environment when the concentration of ammonia and ammonium is higher than safety threshold^12^. Ammonia is poisonous at high concentrations. In addition, as ammonia is lighter than air, it dissipates in the atmosphere easily and its distinctive smell makes unfavorable condition to work in such environment. Moreover, urea is widely used as substrate in the MICP process and because of its agricultural use and being raw material for fertilizer production, economic viability may not be associated with it, in addition to secondary pollutant nature of urea^13^.

In order to solve such problems, the present study focuses on MICP process driven by asparaginase enzyme with commercially available asparagine as substrate. Further, microbially induced sand solidification was studied based on MICP with potential to use as bio-grout. The research has great importance in reducing effect of ammonia on environment without compromising with mechanical and impermeable properties of bio-grout in sand.

## Results

### Asparaginase activity

**Figure 1:**
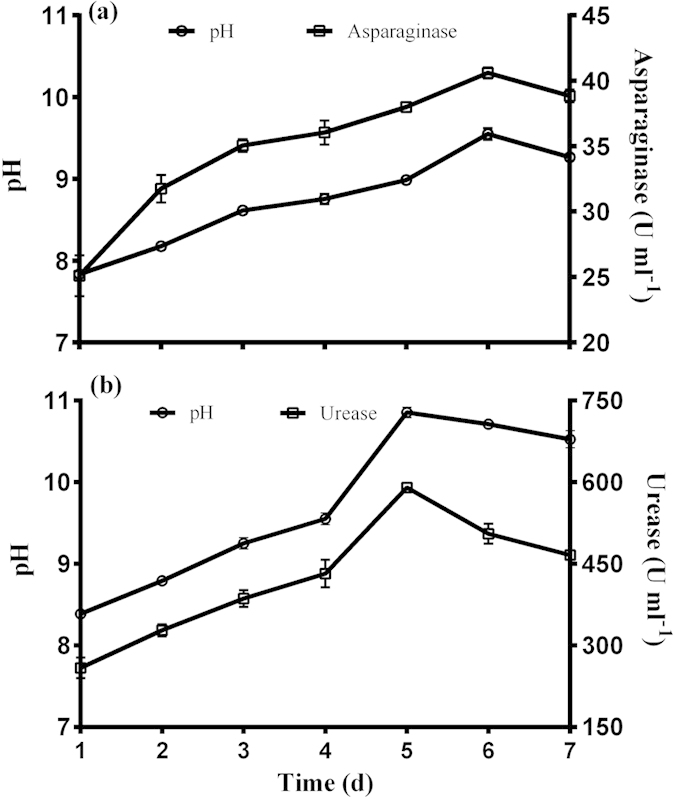
(**a**) pH profile and asparaginase activity, and (**b**) pH profile and urease activity of *B. megaterium* at different time interval.

The present study focused on MICP based on asparaginase, and to compare results with usual enzyme urease for the purpose of biocalcification. At flask level experiments, Nutrient-Asparagine broth (nutrient media supplemented with asparagine) was luxurious for bacterial growth and pH of culture increased up till 9.55 (Fig. 1). The pH increased continuously up till 144 h, and reduced marginally with value 9.3 at 168 h. The asparaginase activity during a period of 7 days ranged from 25.1 to 40.6 U ml^−1^ with maximum activity at 144 h and decreased thereafter.

On other hand, urease activity lead to pH increment till 10.8 at 120 h and at the same time maximum urease production (592 U ml^−1^) was recorded. Urease activity decreased after 120 h.

### Mechanical properties

The UCS (Unconfined Compressive Strength) was obtained at 980 kPa for asparaginase-based bio-grout, compared to that of 1002 kPa due to urease activity; however, it completely failed for grout samples due to lack of calcite precipitation in the absence of bacterial cells. The permeability of asparaginase-based bio-grout was recorded at 2.3 × 10^−7^ m s^−1^ compared to 2.0 × 10^−7^ m s^−1^ of bio-grout produced from urease activity.

### SEM-EDS (Scanning Electron Microscope-Energy Dispersive X-Ray Spectrometer) analysis

**Figure 2:**
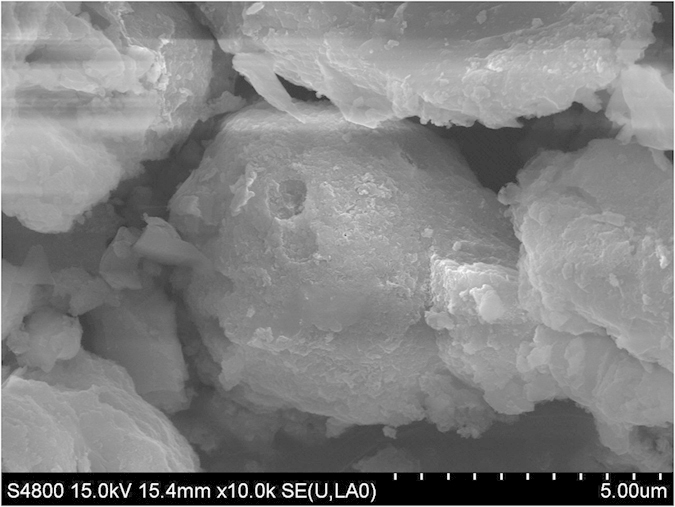
SEM showing imprint of rod-shaped *B. megaterium* precipitating carbonate in bio-grout.

Bio-grout samples presented clear crystal morphology of calcium carbonate nature among particles of sand, when observed under SEM, along with rod-shaped cells of *Bacillus megaterium* (Fig. 2).

**Figure 3:**
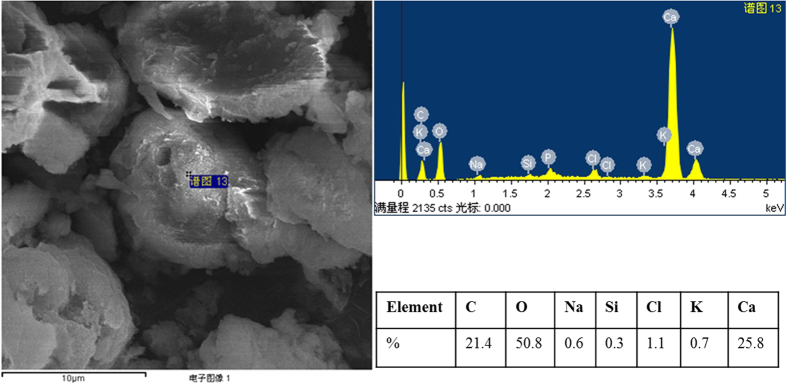
EDS showing high amount of calcium due to *B. megaterium* driven MICP in bio-grout.

In bio-grout, the area chiefly housed by bacterial cells showed high amount of calcium, while higher on calcite crystals precipitated, suggesting calcite precipitation as depicted in EDS spectra (Fig. 3). On other hand, the grout (without bacterial cells) consisted of only media, showed very small amount of calcium coming from constituent of Nutrient-Asparagine media used (data not shown).

### XRD (X-ray Diffraction) and XCT (X-ray Computed Tomography)

**Figure 4:**
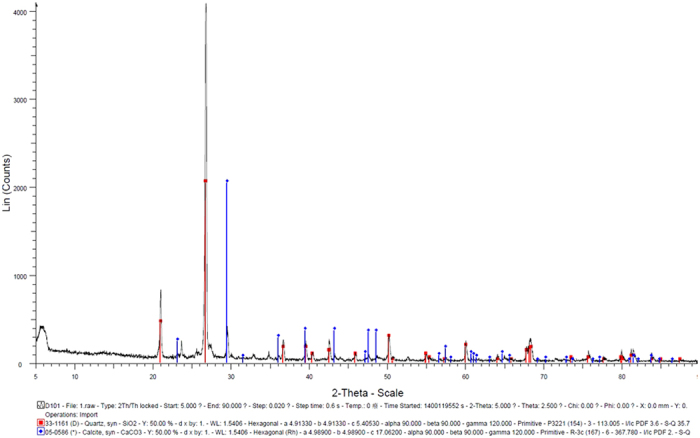
XRD identifying several peaks of calcite precipitated by *B. megaterium* in bio-grout.

The mineralogical composition of precipitated substance in bio-grout was identified using XRD. The bacterial strain used in the present study was able to produce calcium carbonate based on MICP process. X-ray diffractograph showed the mineralogical composition of the precipitated mineral in bio-grout with only observed mineral as calcite (Fig. 4).

**Figure 5:**
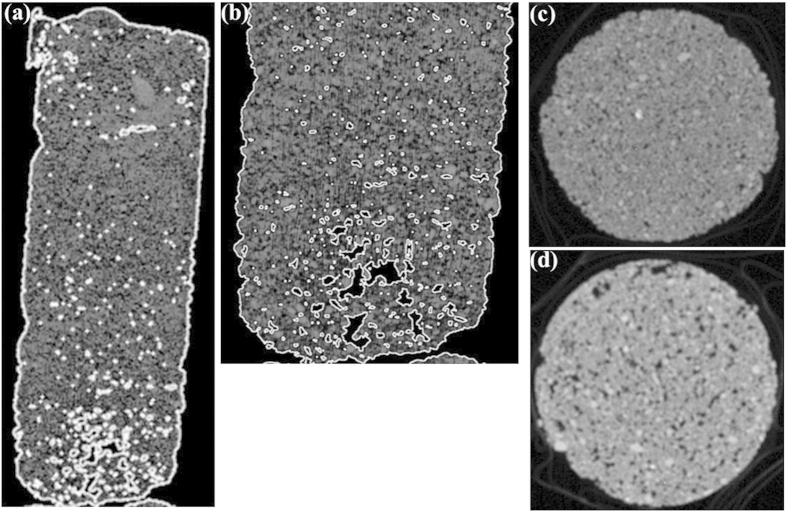
(**a**) Bio-grout as seen under XCT, and (**b**) its closer view from bottom. Cross-section of 2D tomography of bio-grout column from (**c**) top, and (**d**) bottom layer.

To investigate the inner structure and uniformity of bio-grout coming from distribution of the calcite precipitates in the whole column, XCT analysis was performed. The density distribution of the bio-grout at each scanning steps as a result of the XCT is presented in Fig. 5a, while the closer view of the specimen could be seen in Fig. 5b. 2D horizontal slice as cross sections from both top and bottom layers of bio-grout specimen is shown in Fig. 5c,d. The density of the top cross section was higher (Fig. 5c) than that of bottom cross section (Fig. 5d), could be seen clearly due to the presence of air voids. As *B. megaterium* of the present study is aerobic bacterium, higher microbially induced carbonate is expected to precipitate on the top layer compared to the bottom layer.

### Thermogravimetric analysis (TGA)

**Figure 6:**
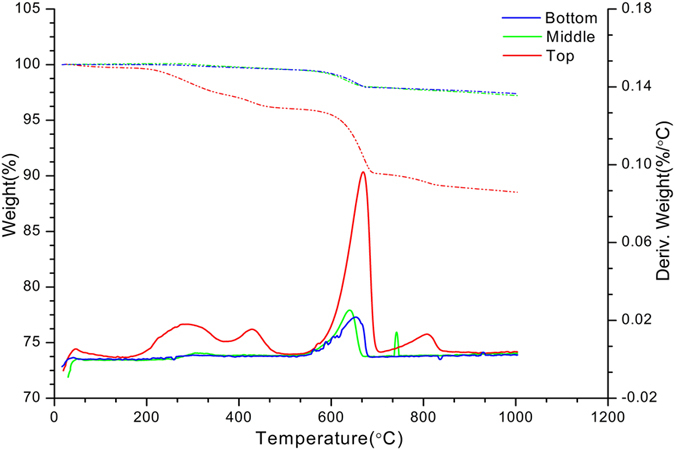
TGA graphs of top, middle and bottom layers of bio-grout specimen (continuous lines represent Weight %, while dotted lines represent Deriv. Weight %/°C).

The mass percentage of calcium carbonate in three layers (top, middle and bottom) of bio-grout calculated via TGA was presented in Fig. 6 in the present study, determined by sharp weight loss due to decarbonation. There were variations in weight losses between different layers of bio-grout as more weight loss was associated with higher calcium carbonate content. The combined large weight loss started at 155 °C and was completed by 1000 °C in top layer of bio-grout; however, started at 405 °C in middle layer of bio-grout (Fig. 6). The bottom layer of bio-grout behaved differently, probably due to low amount of calcite, where weight loss initiated and completed around 1000 °C (Fig. 6). The weight loss due to absorbed water was observed between 0 and 100 °C, while dehydrated CaCl_2_ attributed to weight loss between 100 and 200 °C. The decarbonation or calcium carbonate decomposition led to weight loss between 600 and 900 °C.

## Discussion

The enzyme activities of asparaginase and urease used in the present study measures ammonia liberation, thus provides a comparative feature on ammonia released into the environment. The enzyme asparaginase hydrolyzes asparagine into aspartate and releases ammonium ions (Eq. 9). The hydrolyzed products equilibrate in water to form alanine and bicarbonate ions (Eq. 10) that give rise to an increase in pH and ultimately shift the bicarbonate equilibrium, resulting in the formation of carbonate ions (Eq. 11). Finally, the increased carbonate concentration will induce an increase in supersaturation level leading to CaCO_3_ precipitation around the bacterial cell in the presence of soluble calcium ions (Eqs 12 and 13).


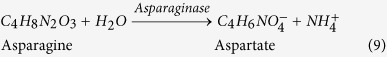



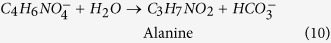



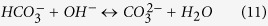



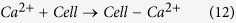



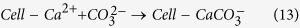


The equation 10 is probable reaction that requires further investigation, if any additional enzyme involved in the process.

The asparaginase activity raised the pH up till 9.5 at 144 h; however, on other hand urease-based MICP resulted in pH increase up to around 11 at 120 h and decreased thereafter. The decreased pH might be due to drop in ammonia production as depicted from a decrease in enzyme activity. Unlike urease activity that decreased after 5 days, asparaginase continued increasing up to 6 days. The maximum release of ammonia, as depicted from enzymatic activities, in the present study was 40.6 U ml^−1^ via asparaginase that was significantly less compared to ammonia production due to urease activity (592 U ml^−1^). In other studies, the maximum urease production by *S. pasteurii* was 412 U ml^−1^ 14, while *B. megaterium* SS3 produced 650 U ml^−1^ of urease^15^. The results of present study suggest that asparaginase activity is preferable over urease in releasing significantly less ammonia into the environment. Further, organic waste could be utilized in future for asparaginase production in the media to make asparaginase-based MICP economic and environment friendly.

To the best of our knowledge, this is the first research where asparaginase rather than urease was explored in detail for MICP. The MICP followed ammonification i.e., the process of amino acid deamination after hydrolysis of protein by heterotrophic bacteria as the pH-increasing reaction^16^.

The results of UCS, and permeability test in bio-grout confirmed that biocalcification or carbonate precipitation by means of bacterial asparaginase is a potential solution for geotechnical problems, as it could induce a gain of compressive strength and at the same time could reduce the permeability. The generation of calcium carbonate could improve the surface density and reduce the permeability of bio-grouts. The results were comparative with urease-based bio-grout, where UCS and impermeability was only marginally higher (Table 1). The MICP process is widely reported to improve the geomechanical properties with a gain of compressive strength^17^.

The bacterial asparaginase activity led to calcite capitation on sand surface, and between sand particles, as shown in SEM (Fig. 2). It has been reported that formation of calcite promotes solidification and increases strength of sandstones or cementitious materials due to MICP^14^,^18^. The MICP process provides well-defined phenomenon in filling of pores in sand rich medium with carbonate crystals^19^. Further, EDS indicated the higher amount of Ca (calcium) in bio-grout that indicated the presence of calcite due to MICP^20^.

The XRD analysis demonstrated that calcium carbonate in the sand biogrout was in the form of calcite, as consistent with TGA results. The similar results were obtained in previous studies where calcite was identified as main biomineral^21^; however, unlike asparaginase based MICP, most of urease driven MICP also resulted in other forms of calcium carbonates such as aragonite and vaterite^14^,^22^. Further studies are required to explain why only calcite was formed as biomineral due to asparaginase activity in sand bio-grout.

As shown under XCT, the inner structure in biogrout was non-uniform and heterogeneous, also observed in previous study^8^. This could be attributed by non-uniform distribution of bacteria and precipitated carbonate in the bio-grout. Tomography is highly desired to locate areas inside the bio-grout columns, as that could not be visually apparent by naked eyes. XCT is recently established as technique to evaluate biocementation in a specimen based on the distribution of calcite precipitation^23^.

The precipitates in bio-grouts were also confirmed to be calcium carbonate by TGA analysis. The results of TGA clearly differentiated calcite content in different layers of bio-grout with amount of calcite decreasing from top to bottom layer. The results were consistent with previous findings where *B. sphaericus* LMG 22257 induced calcium carbonate precipitation in sand^24^. The weight loss before 200 °C, especially clearly shown in top layer of bio-grout was caused by the dehydration of the samples. A sharp weight loss occurred in the temperature range of 600–850 °C in all three layers of bio-grout columns. It was attributed due to calcite decarbonation^25^, while other studies reported weight loss around 650 °C due to decomposition of CaCO_3_^26^,^27^. It has also been reported that calcite produced by biomineralization get decomposed from 600 to 750 °C^18^. The theoretical decomposition reaction for microbially produced calcite is as follows:


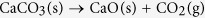


TGA demonstrated a substantial increase in calcium carbonate content in bio-grout compared to grout without bacterial cells, further adds a value upon importance of MICP in the process of grouting.

The calcite amount is not reported to continuous or same throughout the microbial sand column in previous studies. As studied previously, slightly more bacteria were trapped within the first few cm (∼54% in 0–3.8 cm section) of sandstone compared to (∼40% in 3.8–7.5 cm section) due to aerobic nature of *S. pasteurii* induced mineral precipitation^28^. It is also possible that the transport of bacteria might get minimized or limited after calcite precipitation in top layers of bio-grout, leading to lower calcite precipitation in subsequent layers.

In conclusion, the advantage of using asparaginase-based MICP process over urease one is relatively lower production of ammonia that is better for the environment. Additionally, the process doesn’t require urea, which emits secondary pollution. Further research is required to use organic sources of asparagine rather than commercially available to make overall process economic. The asparaginase-based MICP process could reduce huge amount of ammonia released into the environment.

## Materials and Methods

### Materials

The bacterium, *Bacillus megaterium* CGMCC 1.1741 was used in this study. The bacterial strain was maintained on Nutrient Agar medium (pH 8.0). For MICP reactions based on asparaginase activity, Nutrient-Asparagine medium was used with following compositions (per L): 5 g tryptone, 5 g yeast extract and 2 g NaCl, amended with filter sterilized 0.2 M asparagine and 40 mM CaCl_2_ (pH 7.5).

The quartz sand, locally collected, was used as aggregate in the present study with grain size characteristics d_10_ = 150 μm (10% of the grains were smaller than this diameter); d_90_ = 300 μm.

### Asparaginase assay

Asparaginase activity was measured for the confirmation of microbially induced calcite precipitation. The bacterial strain was grown in nutrient broth medium for seed culture development from where 1% of the culture medium was inoculated in Nutrient-Asparagine medium. Asparaginase assay was carried out as per Imada *et al.*^29^. Briefly, 0.5 ml of culture supernatant was collected, added into 0.5 ml of 0.5 M Tris-HCl buffer (pH 8.4) and mixed with 0.5 ml of 0.04 M L-asparagine and milli Q water to a total volume of 2 ml. The mixtures were incubated for 30 min at 37 °C and reaction was stopped by adding 0.5 ml of 1.5 M TCA. To 3.7 ml of milli Q water, 0.1 ml of reaction mixtures was added with 0.2 ml of Nessler’s reagent and the color reaction was allowed to proceed for 20 min before recording absorbance at 450 nm. A standard curve was prepared from solutions of ammonium sulfate as the ammonia source.

For comparison, urease activity was determined in nutrient broth containing filter-sterilized 2% urea and 25 mM CaCl_2_, by measuring the amount of ammonia released from urea as described in Achal *et al.*^14^ One international unit (U) of L-asparaginase or urease is the amount of enzyme, which liberates 1 μmole of ammonia per minute.

### Bio-grout preparation

Bio-grouts were prepared as a result of microbially induced sand solidification in 60 mL plastic syringes column (internal diameter 3.0 cm, length 11.0 cm). The columns were packed with sand up to 10 cm height, after placing a layer of glass wool at the bottom. The columns filled with sand were saturated twice with Tris-HCl buffer that avoids the inclusion of air pockets between sand grains and positioned vertically on clamp. A pump was connected to an injection point at the bottom of the column to regulate the flow rate, as explained in Rong *et al.*^8^.

The bacterial cells grown in Nutrient-Asparagine medium with OD_600_ 1.00 were poured in the sand column at a constant flow rate of 5 mL/min continuously for 10 hours. The columns were left as such and thereafter, Nutrient-Asparagine medium was injected into the column at flow rate of 10 mL/min till no solution leaked out of column. After a week, columns were oven dried at 60 °C for 48 h and bio-grout samples were demoulded for further analyses.

### Strength and permeability of Bio-grout

The bio-grout samples as sandstones were tested to record unconfined compressive strength. The falling head permeability test (as per ASTM D2435-04) was carried out to determine the permeability of asparagine-based bio-grouts and results were compared with urease-based bio-grouts^30^.

### Analytical techniques

Calcium carbonate precipitation in samples was visualized by SEM. Simultaneously, elemental analyses of test piece segments were carried out by using an energy dispersive X-ray spectrometer (EDS) with SEM.

Bio-grout and grout (control) samples were analyzed by XRD to identify cementing components or biominerals. Samples were gently ground in an agate mortar and then classified by size in a 0.037 mm screen aperture with ethyl alcohol^25^. XRD spectra were obtained using Bruker D8 diffractometer with a Cu anode (40 kV and 30 mA) and scanning from 5° to 80° 2θ.

### X-ray computed tomography (XCT)

XCT, which is a forceful non-intrusive technique, examines the internal structure of object depending on the different X-ray absorption of the non-homogeneity materials. According to Beer-Lambert’s law, when monochromatic X-rays are passed through tested material, the final intensities decreases follow this equation:


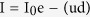


where, I_0_ and I are the initial and the final intensities, respectively. u corresponds the linear absorption coefficient, while d is the length of the sample under test to be measured.

As the absorption can be easily deduced from equation, the collection of absorption measurements over many views allows 2D cross sectional image (CT image) to be mathematically constructed. In this study, bio-grout samples were scanned using German Compact 225 type industrial X-ray computerized tomography. The 2D-images of cross-section (respectively perpendicular to the x-axis, y-axis) in bio-grout samples were reconstructed by VGS studio MAX 2.0 software.

### Thermogravimetry analysis (TGA)

Thermogravimetry analysis was conducted on powdered grout samples. Prior to testing, the samples were kept in a vacuum desiccator for 24 h. The analysis was carried out in TA Instruments incorporated high-resolution thermogravimetric analyzer (series Q600) in a flowing nitrogen atmosphere (80 cm^3^min^−1^), by increasing the temperature from 40 to 1200 °C with a heating rate of 10 °C/min. It is assumed that all weight loss between 700 and 900 °C in TGA was due to calcite decarbonation^16^.

## Additional Information

**How to cite this article**: Li, M. *et al.* Bio-grout based on microbially induced sand solidification by means of asparaginase activity. *Sci. Rep.* **5**, 16128; doi: 10.1038/srep16128 (2015).

## Figures and Tables

**Table 1:** Comparative data of asparaginase and urease in MICP process.

Properties	Asparaginase	Urease
Ammonia release	40.6 U ml^−1^	592 U ml^−1^
UCS (of bio-grout)	980 kPa	1002 kPa
Permeability (in bio-grout)	2.3 × 10^−7^ m s^−1^	2.0 × 10^−7^ m s^−1^
